# Wharton's Jelly Mesenchymal Stem Cell-Derived Extracellular Vesicles Reduce SARS-CoV2-Induced Inflammatory Cytokines Under High Glucose and Uremic Toxin Conditions

**DOI:** 10.1089/scd.2021.0065

**Published:** 2021-08-02

**Authors:** Vuong Cat Khanh, Mizuho Fukushige, Yun Hsuan Chang, Ngo Nhat Hoang, Toshiharu Yamashita, Mana Obata-Yasuoka, Hiromi Hamada, Motoo Osaka, Yuji Hiramatsu, Osamu Ohneda

**Affiliations:** ^1^ Laboratory of Regenerative Medicine and Stem Cell Biology, Departments of University of Tsukuba, Tsukuba, Japan.; ^2^ Obstetrics and Gynecology, University of Tsukuba, Tsukuba, Japan.; ^3^ Cardiovascular Surgery, University of Tsukuba, Tsukuba, Japan.

**Keywords:** SARS-CoV-2, DM, RD, cytokine storm, Calu-3 cells, extracellular vesicles

## Abstract

Cytokine storm is recognized as one of the factors contributing to organ failures and mortality in patients with COVID-19. Due to chronic inflammation, COVID-19 patients with diabetes mellitus (DM) or renal disease (RD) have more severe symptoms and higher mortality. However, the factors that contribute to severe outcomes of COVID-19 patients with DM and RD have received little attention. In an effort to investigate potential treatments for COVID-19, recent research has focused on the immunomodulation functions of mesenchymal stem cells (MSCs). In this study, the correlation between DM and RD and the severity of COVID-19 was examined by a combined approach with a meta-analysis and experimental research. The results of a systematic review and meta-analysis suggested that the odd of mortality in patients with both DM and RD was increased in comparison to those with a single comorbidity. In addition, in the experimental research, the data showed that high glucose and uremic toxins contributed to the induction of cytokine storm in human lung adenocarcinoma epithelial cells (Calu-3 cells) in response to SARS-CoV Peptide Pools. Of note, the incorporation of Wharton's jelly MSC-derived extracellular vesicles (WJ-EVs) into SARS-CoV peptide-induced Calu-3 resulted in a significant decrease in nuclear NF-κB p65 and the downregulation of the cytokine storm under high concentrations of glucose and uremic toxins. This clearly suggests the potential for WJ-EVs to reduce cytokine storm reactions in patients with both chronic inflammation diseases and viral infection.

## Introduction

Since the end of 2019, the pandemic of pneumonia caused by SARS-CoV-2 (COVID-19) has spread worldwide, threatening numerous people's lives [1]. SARS-CoV-2 infection starts when it enters into host body and binds to receptors, including angiotensin-converting enzyme 2 (ACE2) and TMPRSS2, in several types of cells [1]. In lung epithelial cells, this infection activates the cells to release proinflammatory cytokines, recruiting monocytes and macrophages [2,3]. Meanwhile, in alveolar macrophages, SARS-CoV-2 induces the release of proinflammatory cytokines to promote an inflammatory reaction [2,3].

All these reactions result in the release of an abundance of proinflammatory cytokines to promote the immune response against the virus [4]. However, overreaction in the cytokine release causes a cytokine storm in the infected body and dysregulates the inflammatory mechanism leading to cytokine storm, impairment of the organ function, and death [5].

Numerous reports have shown that SARS-CoV-2 infection is associated with severe outcomes and relatively higher mortality in patients with diseases associated with chronic inflammation, such as diabetes mellitus (DM) or renal disease (RD). A study in England showed that the rate of COVID-19-related mortality in patients with type I DM and type II DM was 3.5-times and two times higher, respectively, than that in healthy individuals [6]. In addition, a study from the United States revealed that the mortality rate of COVID-19 patients with DM was four times that of patients without DM [7]_._ Moreover, a recent meta-analysis conducted by Huang et al. reported that the risk ratio of COVID-19-related mortality was more than double among DM patients [8]. Of note, a recent study suggests that together with the cytokine storm induced by SARS-CoV-2 infection, the chronic inflammation in patients with DM might contribute to the more severe outcomes of the dysregulated inflammatory reaction in COVID-19 [9].

In addition to DM, RD is also recognized as a risk factor for severe outcomes and mortality in patients with COVID-19. An analysis of data from more than 17 million people in UK suggested that patients with chronic RD are at higher risk than patients with other diseases related to the heart or lung [10]. Moreover, patients with RD also show numerous comorbidities, such as hypertension and DM, which contribute to poor outcomes in individuals with COVID-19 infection [11–13]. Although the correlation between chronic diseases, such as DM or RD, with the increased mortality of patients with COVID-19 has been examined in numerous studies, the combination effects of both DM and RD on the outcomes of COVID-19 have received little attention.

Mesenchymal stem cells (MSCs) are a type of multipotent stem cell; they exist in different types of tissue, including bone marrow, adipose tissue, and Wharton's jelly from umbilical cord [14]. The application of MSCs in the treatment of infectious diseases has been reported due to their immunomodulatory functions, which attenuate inflammatory reactions [14]. Recently, a research group reported that transplantation of MSCs to COVID-19 patients promoted tissue repair in the lung, and might be involved in the regulation of excessive inflammatory reaction [15]. Interestingly, another study reported that MSCs show no expression of ACE2 or TMPRESS2, and are resistant to SARS-CoV-2 infection under steady-state, inflammatory conditions, and in the presence of SARS-CoV-2 infected cells [16].

Extensive research has shown that the functions of MSCs are predominantly performed through paracrine pathways by soluble mediators, such as growth factors, cytokines, chemokines, and extracellular vesicles (EVs) [17]. EVs are a heterogeneous group of membrane vesicles that includes exosomes and microvesicles, which are released from MSCs and which contain various molecules, such as mRNA, miRNAs, proteins, and lipids [18]. Recent research has demonstrated that, in addition to their function as a tool of cell-cell communication, EVs from MSCs also possess an immunomodulation ability that is similar to that of the parental MSCs, suggesting that EVs might be applied in the treatment of several autoimmune diseases and infectious diseases [19]. In addition, the cargo of EVs from normal MSCs has been proven to prevent virus replication [20]. Notably, a recent study reported that the injection of exosomes from bone marrow-derived MSCs enhanced the improved outcomes of patients with COVID-19 [21]. This evidence suggests that the EVs from MSCs might be a promising candidate for the treatment of COVID-19 patients with more stable, effective, and safer values than the parental MSCs themselves.

In this study, a systemic review and meta-analysis were performed to clarify the role of DM and RD in the severe outcomes of patients with COVID-19, and then experimental research was conducted to investigate the inflammatory reactions of Calu-3 cells in response to the induction of SARS-CoV-2 Peptide Pools under high glucose and uremic toxin concentrations. In addition, the effects of EVs from MSCs on the downregulation of inflammatory reactions in Calu-3 cells by SARS-CoV-2 Peptide Pools were also examined to study the potential of EVs for the treatment of COVID-19.

## Materials and Methods

### Statement

All experiments and methods included in this study were performed according to the amended Declaration of Helsinki and were approved by the Ethics Committee of the University of Tsukuba. The collection of human samples, such as adipose tissue and umbilical cords, and relevant experiments were conducted after obtaining the informed consent of the donors.

### Systematic review and meta-analysis

An electronic literature search was conducted using PubMed (https://pubmed.ncbi.nlm.nih.gov) and Web of Science (https://apps.webofknowledge.com). The search terms were (COVID-19 OR SARS-CoV-2) AND diabet* AND (kidney OR renal). This search was completed on January 5, 2021. Duplicate articles were removed, after which the titles and abstracts were reviewed. The full texts of the relevant articles were sourced through PubMed, Web of Science, and Google Scholar (scholar.google.com). The titles and abstract and/or full text of all articles were reviewed by at least two independent reviewers out of three authors (K.V., N.H., and M.F.).

A study was considered eligible if it met all of the following inclusion criteria: (1) involved human participants, (2) was based on SARS-CoV-2 infection, (3) reported the severity and/or mortality of COVID-19 in patients with RD (including both chronic RD and acute RD) and DM (including both Type 1 and Type 2 DM), and (4) in comparison that in RD patients with/without DM or DM patients with/without RD. Studies were excluded based on the following exclusion criteria: (1) use of non-human subjects, (2) performance in vitro, (3) involved fewer than 10 participants (eg, a clinical case report), (4) review article or protocol article, and (5) participants were specially selected based on comorbidities other than RD and/or DM.

A meta-analysis was conducted to calculate the individual and pooled odds ratios (ORs) and 95% confidence intervals. The meta-analysis was performed using the RevMan 5 software program (Version 5.4, The Cochrane Collaboration 2020, Copenhagen, Denmark).

### MSC isolation and culture

Human adipose tissue was obtained from nondiabetic non-RD donors (*n* = 4, male, average age: 70 years), who were undergoing procedures in the Department of Cardiovascular Surgery, University of Tsukuba Hospital, Tsukuba, Japan. The isolation of MSCs was performed according to the previously described method [22].

Briefly, adipose tissues were minced into pieces of <3 mm in size, and then treated with 0.1% collagenase (Nitta Gelatin, Osaka, Japan) in PBS and 20% fetal bovine serum (FBS; Hyclon, South Logan, UT) for 45 min at 37°C, and filtered through a 100-μm nylon mesh (BD Biosciences, San Jose, CA). Then, samples were centrifuged at 1600 rpm in 7 min and washed to remove the collagenase solution. The isolated cells were cultured in MSC culture medium containing Iscove's modified Dulbecco's medium (IMDM) (Thermo Fisher Scientific, Carlsbad, CA) with 10% FBS, 2 mg/mL l-glutamine (Thermo Fisher Scientific), 5 ng/mL human basic-FGF (Peprotech, London, United Kingdom), and 0.1% (v/v) penicillin–streptomycin (100 U/mL penicillin and 0.1 mg/mL streptomycin; Thermo Fisher Scientific) at 37°C in 5% CO_2_ and a humidified atmosphere.

Human umbilical cords were obtained from nondiabetic non-RD donors (*n* = 4, female, average age: 32), who were undergoing cesarean section in the Department of Obstetrics and Gynecology, University of Tsukuba Hospital.

Human umbilical cords were cut to expose the blood vessels and Wharton's jelly. Then, blood vessels were removed and Wharton's jelly was collected and cut into 1–2 mm pieces. After that, the Wharton's jelly pieces were incubated with Trypsin solution at 37°C for 30 min in a 5% CO_2_ incubator for partial digestion. After incubation, an equal volume of MSC culture medium was added and incubated for 3 min, and then 15–20 digested tissue pieces were carefully plated on a culture dish containing MSC culture medium at 37°C under 5% CO_2_ and a humidified atmosphere. After 3–5 days of incubation, the appearance of isolated WJ-MSCs was confirmed and the medium was replaced with fresh medium. All MSCs used for the experiments of this study were at passage 3–8.

### Human lung adenocarcinoma epithelial cell culture and induction with SARS-CoV-2 Peptide Pools

The Calu-3 cell line (LGC Standards, Cat No. ATCC-HTB-55, American Type Culture Collection-ATCC, Manassas, VA) was kindly provided by the Department of Human Pathology, University of Tsukuba. Calu-3 cells were cultured in Eagle's Minimum Essential Medium (EMEM; Thermo Fisher Scientific) medium containing 10% FBS with 1% penicillin/streptomycin at 37°C under 5% CO_2_ and a humidified atmosphere. The medium was changed thrice per week. Upon reaching 80% confluence, the cells were harvested and subcultivated at a ratio of 1:5. A number of 10^5^ Calu-3 cells were induced by SARS-CoV-2 pepTivator Peptide Pools Prot_S (Miltenyi Biotec, Bergisch Gladbach, Germany) at a concentration of 6 pmol for 24 h before collection for further analyses.

### A fluorescence activated cell sorting analysis

To examine the surface expression of ACE2 or TMPRSS2, a number of 5 × 10^5^ Calu-3 cells in 200 μL PBS containing 3% FBS and 1% sodium azide were incubated with primary antibody, including 5 μL rabbit anti-ACE2 antibody (GeneTex, Zeeland, MI; GTX101395) or 2 μL rabbit anti-TMPRSS2 antibody (GeneTex; GTX81494), for 30 min at 4°C in the dark. The cells were washed thrice by cold PBS and resuspended in 200 μL PBS. The cells were incubated with secondary antibody goat anti-rabbit IgG DyLight488 (GeneTex; GTX213110-04, dilution rate 1:100) antibody for 30 min at 4°C in the dark, then washed thrice by cold PBS, resuspended in 300 μL PBS containing 3% FBS and 1% sodium azide, and analyzed by a cell sorter (SH300S; Sony Technology, San Jose, CA). The isotype rabbit IgG was used as the negative control (Genetex; GTX35035). The relative surface expression was quantified by means of fluorescent intensity value subtracted with isotype control value.

To examine the surface markers of MSCs, a number of × 10^5^ Calu-3 cells in 200 μL PBS containing 3% FBS were incubated with primary antibodies for 30 min at 4°C in the dark, including FITC-labeled anti-CD90 (BioLegend, San Diego, CA; 328107), PE-labeled anti-CD105 (BioLegend; 323206), PE-labeled anti-CD73 (BD Biosciences, San Diego, CA, 550257), PE-labeled anti-CD31 (BioLegend; 303106), and APC–labeled anti-CD45 (BD Biosciences; 555485). The isotype controls include APC-labeled anti-IgG1 (555751; BD Biosciences), PE-labeled anti-IgG1 (555749; BD Biosciences), and FITC-labeled anti-IgG1 (555748; BD Biosciences). Then, cells were washed with cold PBS and resuspended in 300 μL PBS containing 3% FBS and analyzed by a cell sorter (SH300S; Sony Technology).

### Proliferation assay

Calu-3 cells were seeded at 10^5^ cells/well in a 24-well plate in EMEM containing 10% FBS with 1% penicillin/streptomycin at 37°C under 5% CO_2_ and a humidified atmosphere. The cells were harvested and live cell numbers were counted every day by staining with trypan blue solution (Nacalai Tesque, Kyoto, Japan) using a hemocytometer.

### Gene expression analysis

To examine the gene expression, total RNA was collected from Calu-3 cells and isolated by Sepasol-RNA I Super G (Nacalai Tesque) in accordance with the manufacturer's protocol. Total RNA (1 μg) was reverse transcribed using an RT-PCR kit (Toyobo, Osaka, Japan). cDNA was analyzed using a GeneAmp 7500Fast Real-Time PCR System (Applied Biosystems) using SYBR green reagent (Toyobo). The expression levels of the target genes were analyzed using the ΔΔCt method. The sequences of the primer sets used for the PCR are shown in Table 1.

**Table 1.** The Primer Sets Used for Quantitative Polymerase Chain Reaction

Gene	Forward primer	Reverse primer
*tnfα*	TCCTTCAGACACCCTCAACC	AGGCCCCAGTTTGAATTCTT
*il6*	TACCCCCAGGAGAAGATTCC	TTTTCTGCCAGTGCCTCTTT
*il1β*	GGGCCTCAAGGAAAAGAATC	TTCTGCTTGAGAGGTGCTGA
*ifnγ*	GAGTGTGGAGACCATCAAGGAAG	TGCTTTGCGTTGGACATTCAAGTC
*ace2*	GGGATCAGAGATCGGAAGAAGAAA	AGGAGGTCTGAACATCATCAGTG
*tmprss2*	AATCGGTGTGTTCGCCTCTAC	CGTAGTTCTCGTTCCAGTCGT
*β-actin*	GTGCGTGACATTAAGGAGAAGCTGTGC	GTACTTGCGCTCCAGGAGGAGCAATGAT

### Magnetic Luminex performance assay

A number of 10^5^ Calu-3 cells were induced by 6pmol SARS-CoV-2 pepTivator Peptide Pools Prot_S (Miltenyi Biotec) at a concentration of 6 pmol for 24 h, followed by treatment with EVs for a further 24 h before the collection of conditioned medium. The conditioned media were centrifuged at 16,000 *g* for 4 min before use in a Magnetic Luminex assay to measure the inflammatory cytokine concentration using a Human High Sensitivity Cytokine Premixed Kit A (R&D Systems, Minneapolis, MN) according to the manufacturer's instructions.

Briefly, 100 μL of standard or conditioned medium was added to each well of a 96-well plate, and then Microparticle Cocktail was added, followed by incubation for 3 h at room temperature and washing with Wash Buffer. Next, the mixture was incubated with Biotin-Antibody Cocktail, including a mixture of anti-TNFα, anti-IL6, anti-IL1β, and anti-IFNγ for 1 h at room temperature, followed by incubation with Streptavidin-PE for 30 min at room temperature. After washing, the data were analyzed using a Luminex^®^ 200 Multiplexing Instrument (Merck Millipore, Burlington, MA). The culture medium, EMEM containing 10% FBS with 1% penicillin/streptomycin, was used as the blank sample. The concentration of each inflammatory cytokine ([X]) was calculated as follows:

$$\begin{array}{cc} \left[X\right] & =\left[X\right]measuredinexperimentsample\\ & -\left[X\right]measuredinblanksample \end{array}$$

### Co-culture of MSCs and Calu-3

A number of 10^5^ Calu-3 cells were seeded in the lower chamber of an 8-μm pore Transwell (Corning Incorporated, New York, NY) containing 500 μL of completed culture. Cells were maintained at 37°C under 5% CO_2_ for 6 h to allow cell attachment, and then induced by 6 pmol SARS-CoV-2 Peptide Pools Prot_S. After that, 10^5^ MSCs were seeded into the upper chamber of the Transwell and the co-culture was maintained at 37°C under a 5% CO_2_ atmosphere for a further 24 h. At the end of co-culture, Calu-3 cells were collected and the gene analysis was performed.

### EV isolation

The medium of subconfluent AT-MSCs or WJ-MSCs was changed to Iscove's modified Dulbecco's medium (IMDM) with 1% penicillin/streptomycin and 0.25% EV-depleted FBS (Thermo Fisher Scientific). After 24 h, the supernatants were collected and centrifuged at 1000 rpm for 5 min, and then centrifuged at 2100 rpm for 20 min to remove cell debris from the medium. Cell-free supernatants were ultracentrifuged at 100,000 rpm for 70 min at 4°C, and the pellets were stained with PKH26 (PKH26 linker; Sigma-Aldrich, St. Louis, MO) for 5 min at room temperature. After that, the washing step was performed twice by adding PBS with 0.25% EV-depleted FBS (Thermo Fisher Scientific) and ultracentrifuged at the same conditions. After washing, pellets of EVs were collected.

EVs then were characterized by quantification of the protein concentration using the Bradford method (Bio-Rad, Hercules, CA), size measurement using a particle size analyzer (FDLS3000; Shimadzu Corporation, Kyoto, Japan), and a morphology analysis by transmission electron microscopy (JEM-1400Flash; JEOL Ltd., Tokyo, Japan).

### Incorporation of EVs to Calu-3 cells

A number of 10^5^ Calu-3 cells/well were seeded in a 12-well plate and maintained at 37°C under 5% CO_2_ for 6 h to allow cell attachment, and then induced by 6 pmol SARS-CoV-2 Peptide Pools Prot_S for 24 h. After that, 20 μg PKH-26-labeled EVs was added in the Calu-3 cells for another 24 h. The incorporation of PKH-26-labeled EVs to Calu-3 cells was examined by a flow cytometry (Sony Biotechnology, San Jose, CA).

### Western blotting

Nuclear cell extract (20 μg in each well) was electrophoretically separated on 7.5% SDS-polyacrylamide gels and electrotransferred to polyvinylidene difluoride membranes (Millipore). Membranes were blocked with 5% skim milk, and 1% FBS in TBS-T (Tris-buffered saline containing 0.1% Tween 20) for 1 h at room temperature, and then incubated with primary antibodies Rabbit anti-NF-κB p65 (Proteintech, Rosemont, IL; 10745-1-AP) at 1:1000 dilution at 4°C overnight. Goat anti-Lamin B antibody (SantaCruz Biotechnology, Dallas, TX, sc-6216) at 1:1000 dilution was used as the antibody for the internal control.

For EVs' markers, total protein extracted from EVs (20 μg in each well) was separated by electrophoresis followed by the transfer to PVDF membranes. After blocking, membranes were incubated with primary antibodies, including rabbit anti-CD63 (Cusabio Tachnology LLC, Houston, TX; CSB-PA006039) and rabbit anti-TSG101 (Cusabio Technology LLC; CSB-PA060017), at 1:1000 dilution. After extensive washing with TBS-T, the membranes were incubated with the following Horseradish peroxidase (HRP)-conjugated secondary antibodies: HRP-conjugated rabbit anti-goat IgG or HRP-conjugated goat anti-rabbit IgG (Thermo Fisher Scientific) at 1:10,000 dilution, and positive signals were analyzed by a luminescence imager (Image Quant LAS4000; GE Health Care, Little Chalfont, United Kingdom) using chemiluminescence reagents (Merck Millipore). The expression level of proteins was analyzed using the ImageJ software program [23] (NIH, Bethesda, MD).

### Statistical analyses

Data were statistically analyzed by the Mann–Whitney U-test using the GraphPad Prism 5 software program (GraphPad Software, San Diego, CA). Data are presented as the mean ± standard deviation. *P* values of <0.05 were considered to indicate statistical significance.

## Results

### The combination of DM and RD increased the odds of mortality in patients with COVID-19 according to a meta-analysis

**FIG. 1.**
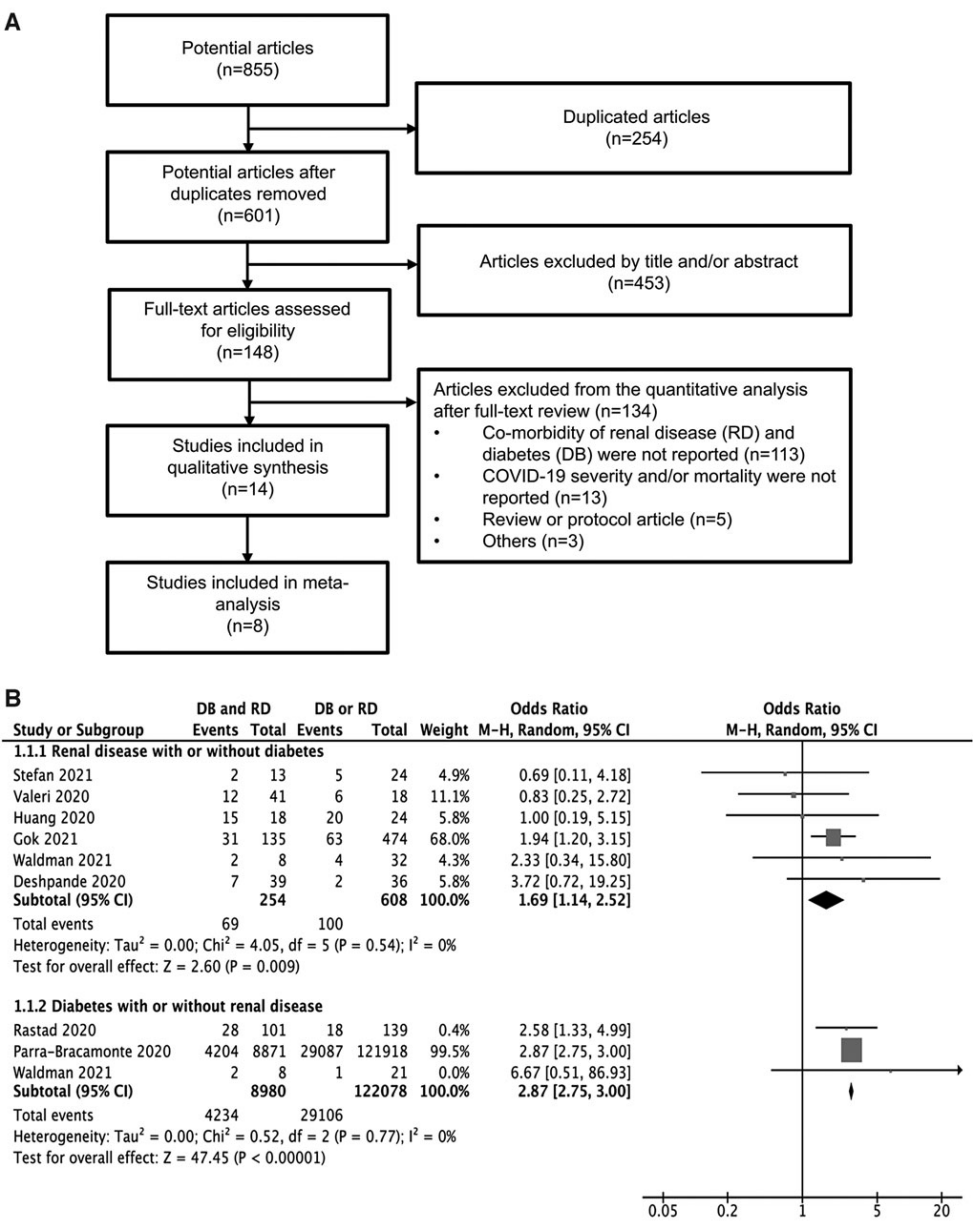
A systematic review and meta-analysis of the correlation between DM and RD and the risk of mortality in patients with COVID-19. **(A)** A flow diagram of the systematic review. A flow diagram of the number of articles identified and examined at each stage of the systematic review. A total of eight articles published from 2020 to 2021 met all inclusion criteria and were included in the meta-analysis. **(B)** Odds ratios for COVID-19-related mortality in patients with RD, DM, or RD and DM. DM, diabetes mellitus; RD, renal disease.

An electronic literature search identified 853 articles. Two articles were identified from other sources. From these 855 articles, the full text of 148 articles was screened. A total of 14 eligible articles published from 2020 to 2021 were included in the qualitative synthesis. Eight of these articles were included in the meta-analysis (Fig. 1A).

The results of the meta-analysis showed that the OR for mortality in RD patients with DM was 1.69 times higher compared with RD patients without DM (OR = 1.69, 95% CI = 1.14–2.52, *P* < 0.01, Fig. 1B). Similarly, the results also showed that the OR for mortality of DM patients with RD was 2.75 times higher compared with DM patients without RD (OR = 2.75, 95% CI = 2.75–3.00, *P* < 0.01, Fig. 1B). Taken together, these results showed that in comparison to a comorbidity of DM or RD, the combination of DM and RD highly increased the odds of mortality in patients with COVID-19.

### SARS-CoV-2 Peptide Pools induced the cytokine storm of human lung adenocarcinoma epithelial cells, Calu-3 cells

Because both DM and RD have been reported as factors contributing to chronic inflammation [9,24], based on the results of the meta-analysis, we next considered whether COVID-19 patients with DM and RD have more severe inflammatory cytokine storm responses to SARS-CoV2 infection in comparison to those without any comorbidity or those with DM or RD as a single comorbidity.

**FIG. 2.**
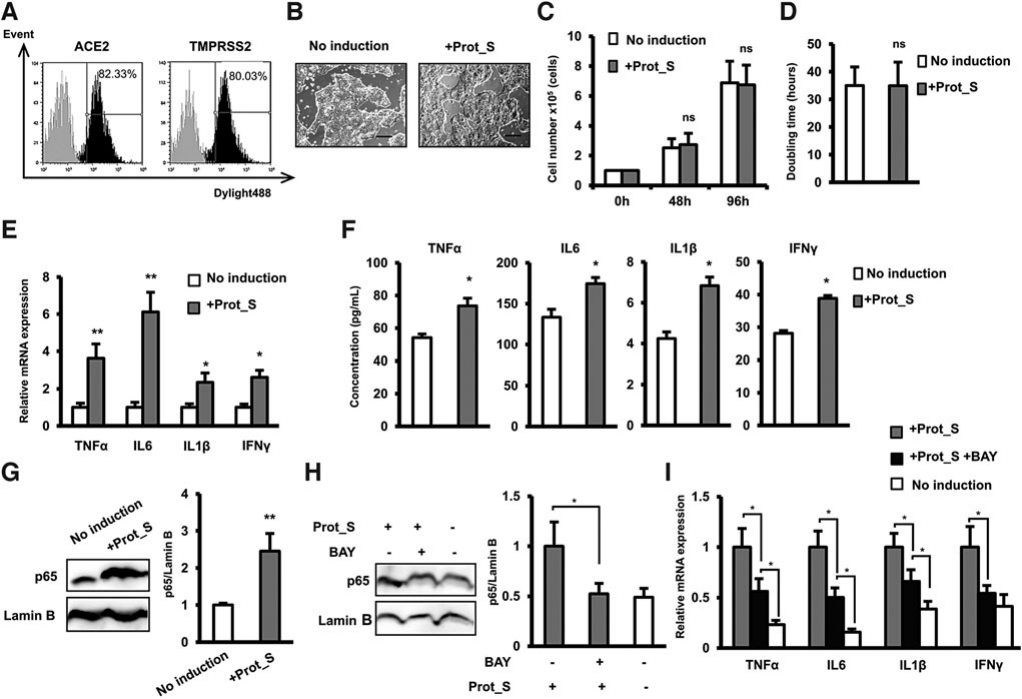
SARS-CoV-2 Peptide Pools induced cytokine storm in Calu-3 cells. **(A)** The surface expression of ACE2 and TMPRSS2 in Calu-3 cells examined by flow cytometry. (**B)** The morphology of Calu-3 cells under a microscope at 40 × magnification. Bars indicated 100 μm. **(C)** The proliferation of Calu-3 cells. **(D)** Doubling time of Calu-3 (*n* = 3). **(E)** The inflammatory cytokine gene expression in Calu-3 cells (*n* = 3). **(F)** The inflammatory cytokine secretion in Calu-3 cells (*n* = 4). **(G)** The nuclear p65 protein expression in Calu-3 cells. **(H)** The nuclear p65 protein expression in Calu-3 cells in the presence of NF-κB inhibitor BAY11-7082. I. The inflammatory cytokine gene expression in Calu-3 cells in the presence of NF-κB inhibitor. The data represent the mean ± SD. **P < 0.01, *P < 0.05, ns: no significance. The experiments were performed in triplicate.

Previous report showed that human lung adenocarcinoma epithelial cell (Calu-3 cell) response to respiratory syncytial virus is similar to the normal human bronchial epithelial cells, which is a suitable in vitro model to study the host responses to respiratory syncytial virus infection [25]. In addition, Calu-3 cells, which were reported to be susceptible to SARS-CoV-2 infection [26,27], were also used as a model of lung epithelial cells' responses to screen antiviral drugs to treat SARS-CoV2 [28]. Therefore, we next performed experimental research on the inflammatory reactions of Calu-3 cells in response to SARS-CoV-2 PepTivator Peptide Pools Prot_S, a spike glycoprotein that is responsible for the recognition and binding of SARS-CoV-2 to the host cells, under high glucose and uremic toxin concentrations, which are reported as consequences of DM [29] and RD [30], respectively. After the surface expressions of ACE2 and TMPRSS2, which are reported as the receptor of SARS-CoV2 spike protein S, in Calu-3 cells were confirmed (Fig. 2A), the responses of Calu-3 cells to Prot_S were examined.

As shown in Fig. 2B, the induction of Prot_S showed no alteration of the cell morphology, with Calu-3 cells maintaining their epithelial morphology after 96 h of induction. In addition, none of the SARS-CoV-2 Peptide Pools showed any effect on the proliferation rate of the Calu-3 cell line, the average doubling time of which is ∼33 h (Fig. 2C, D).

Viral infection is associated with the induction of a cytokine storm; thus, we next examine the expression of inflammatory cytokines in Calu-3 cells after induction by Prot_S. As expected, after 24 h of incubation, Calu-3 cells induced by Prot_S showed the significant upregulation of inflammatory cytokines, including TNFα, IL6, IL1β, and IFNγ (TNFα: 3.6-fold increase, IL6: 6.1-fold increase, IL1β: 2.3-fold increase, and IFNγ: 2.6-fold increase, *n* = 3, *P < 0.05, **P < 0.01, Fig. 2E). Consistent with the mRNA expression, the induction of Pro_S was associated with the increased secretion of inflammatory cytokines, including TNFα, IL6, IL1β, and IFNγ (TNFα: 1.4-fold increase, IL6: 1.3-fold increase, IL1β: 1.6-fold increase, and IFNγ: 1.4-fold increase, *n* = 4, *P* < 0.05, Fig. 2F). The NF-κB signal transduction pathway is recognized as a mediator of the proinflammatory gene expression [31]. Therefore, we next examined the involvement of the NF-κB pathway in the induction of Calu-3 by Prot_S. Remarkably, the results showed that in response to Prot_S, Calu-3 cells showed an increase of nuclear p65 (2.45-fold increase, *n* = 3, *P* < 0.01, Fig. 2G). In addition, treatment of Prot_S-induced Calu-3 cells with NF-κB inhibitor BAY 11-7082 (Sigma-Aldrich; BAY) showed the decrease of nuclear p65 (Fig. 2H), which resulted in the downregulation of inflammatory cytokines (Fig. 2I), suggesting that the upregulation of p65 is involved in the induction of a SARS-CoV2 peptide-induced cytokine storm in Calu-3 cells.

Taken together, these data indicated that Calu-3 cells showed the upregulation of inflammatory cytokines and NF-κB p65 under induction by SARS-CoV-2 Peptide Pools.

### MSC-derived EVs reduced the SARS-CoV-2 peptide-upregulated inflammatory cytokines in Calu-3 cells

**FIG. 3.**
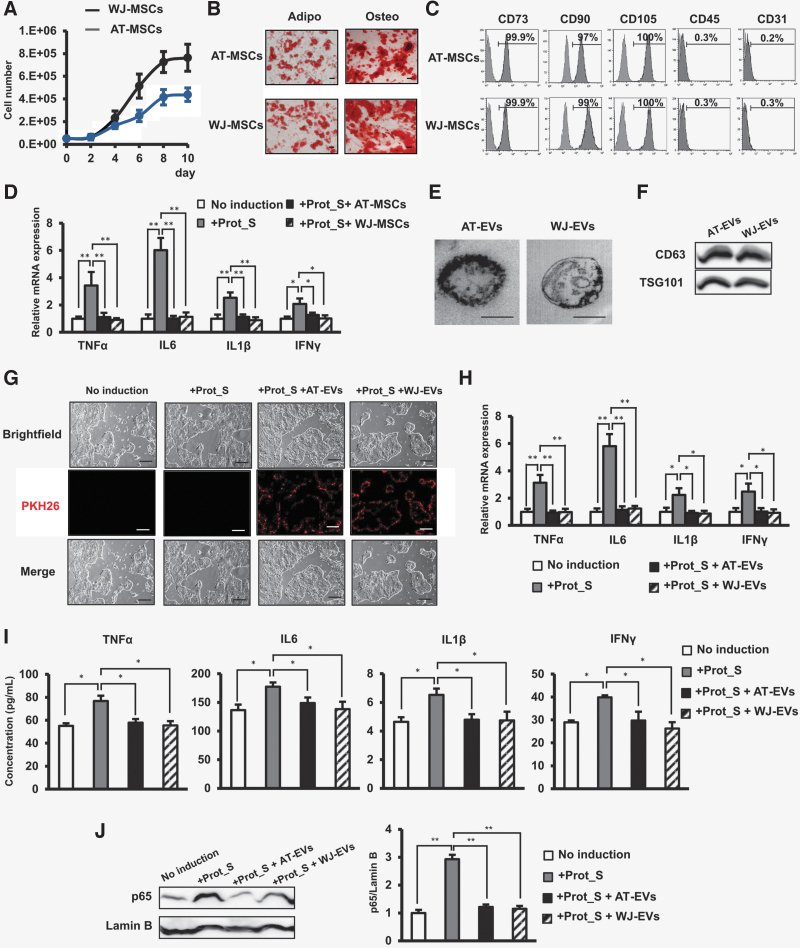
MSC-EVs reduced the upregulation of the cytokine storm in Calu-3 cells induced by SARS-CoV-2 Peptide Pools. **(A)** Growth curve of MSCs. **(B)** Adipogenic and osteogenic differentiation of MSCs after 20 days. Adipo: Adipogenic differentiation, Osteo: Osteogenic differentiation. The photos were taken under a microscope at 200 × magnification. Bars indicated 100 μm. **(C)** Cell surface markers of MSCs. **(D)** The inflammatory cytokine expression in Calu-3 cell co-culture with MSCs. Four independent cell lines of MSCs were used in the above experiments (*n* = 4). **(E)** The morphology of EVs under a transmission electron microscopy. Bars indicated 100 nm. **(F)** The marker expression of EVs. **(G)** The incorporation of EVs into human lung epithelial cells after 24 h. The photos were taken under a microscope at 40 × magnification. Bars indicated 100 μm. **(H)** The inflammatory cytokine expression in Calu-3 cells incorporated with EVs. **(I)** The inflammatory cytokine secretion in Calu-3 cells incorporated with EVs (*n* = 4). **(J)** The nuclear p65 protein expression in Calu-3 cells incorporated with EVs. EVs isolated from four independent cell lines of MSCs were used in the above experiments (*n* = 4). The data represent the mean ± SD. **P < 0.01, *P < 0.05, ns: no significance. The experiments were performed in triplicate. MSC, mesenchymal stem cell; EV, extracellular vesicle.

Recent clinical trials have reported the potential application of MSCs, as a result of their immune-modulatory functions, in the treatment of SARS-CoV-2 infection [15]. Therefore, we next examined how MSCs derived from adipose tissue (AT-MSCs) and Wharton's jelly (WJ-MSCs) regulate the SARS-CoV-2 peptide-induced inflammatory cytokines in Calu-3 cells. AT-MSCs and WJ-MSCs were characterized by their high proliferation rate (Fig. 3A), the ability to differentiate into adipocytes and osteocytes (Fig. 3B), and the expression of MSC markers: they were positive for CD90, CD73, and CD105, and negative for CD45 and CD31 (Fig. 3C). Remarkably, co-culture of Calu-3 cells with either AT-MSCs or WJ-MSCs in a Transwell co-culture system reduced the upregulation of inflammatory cytokines of Calu-3 cells, which was induced by Prot_S to the normal state, similar to Calu-3 cells without induction by Prot_S (Fig. 3D).

Based on extensive research on the functions of MSCs, it is reported that MSCs exert their paracrine functions through soluble mediators, including EVs [32]. In addition, it is reported that exosomes from bone marrow-derived MSCs showed the capacity to restore oxygenation, downregulate the cytokine storm, and reconstitute the immunity of patients with COVID-19 [21]. In this study, we examined whether or not EVs derived from AT-MSCs (AT-EVs) and WJ-MSCs (WJ-EVs) also showed recovery effects on SARS-CoV-2 PepTivator-induced cells. First, the analysis of EVs under a transmission electron microscope showed that both AT-EVs and WJ-EVs exhibited a round shape with a diameter of 200 nm (Fig. 3E). In addition, both AT-EVs and WJ-EVs showed the expression of EV markers, which were positive for CD63 and TSG101 (Fig. 3F).

Next, the PKH-26-red-labeled AT-EVs or WJ-EVs were incorporated into the Prot_S-induced Calu-3 cells. The incorporation of AT-EVs or WJ-EVs into the Calu-3 cells was confirmed by the PKH-26-red signals under a fluorescence microscope (Fig. 3G). As a result, the incorporation of AT-EVs or WJ-EVs significantly reduced the expression and secretion of inflammatory cytokines, including TNFα, IL6, IL1β, and IFNγ, in Prot_S-induced Calu-3 cells to normal levels, similar to those in Calu-3 cells without induction (Fig. 3H, I). Moreover, treatment with AT-EVs or WJ-EVs resulted in a significant decrease of NF-κB p65 in the nucleus of Prot_S-induced Calu-3 cells (Fig. 3J).

Taken together, these data suggested AT-EVs or WJ-EVs possess the ability to reduce the cytokine storm and the level of nuclear NF-κB p65 in Calu-3 cells after induction by SARS-CoV-2 Peptide.

### High glucose and uremic toxin concentrations induced the expression of inflammatory cytokines in Calu-3 cells

Recent studies reported the higher mortality rate and severity of COVID-19 in patients with DM or RD [6–13]. In addition, our meta-analysis results showed that the risk ratio for COVID-19-related mortality was higher in COVID-19 patients with DM or RD as a single comorbidity. DM is associated with increased blood glucose concentrations [33], while RD is associated with the accumulation of high concentrations of uremic toxins [30], suggesting the involvement of one of the factors associated with the induction of inflammatory cytokines [34,35]. Therefore, in this study, we next examined how high glucose or uremic toxin concentrations affect the cytokine storm induction of cells induced by SARS-CoV2.

**FIG. 4.**
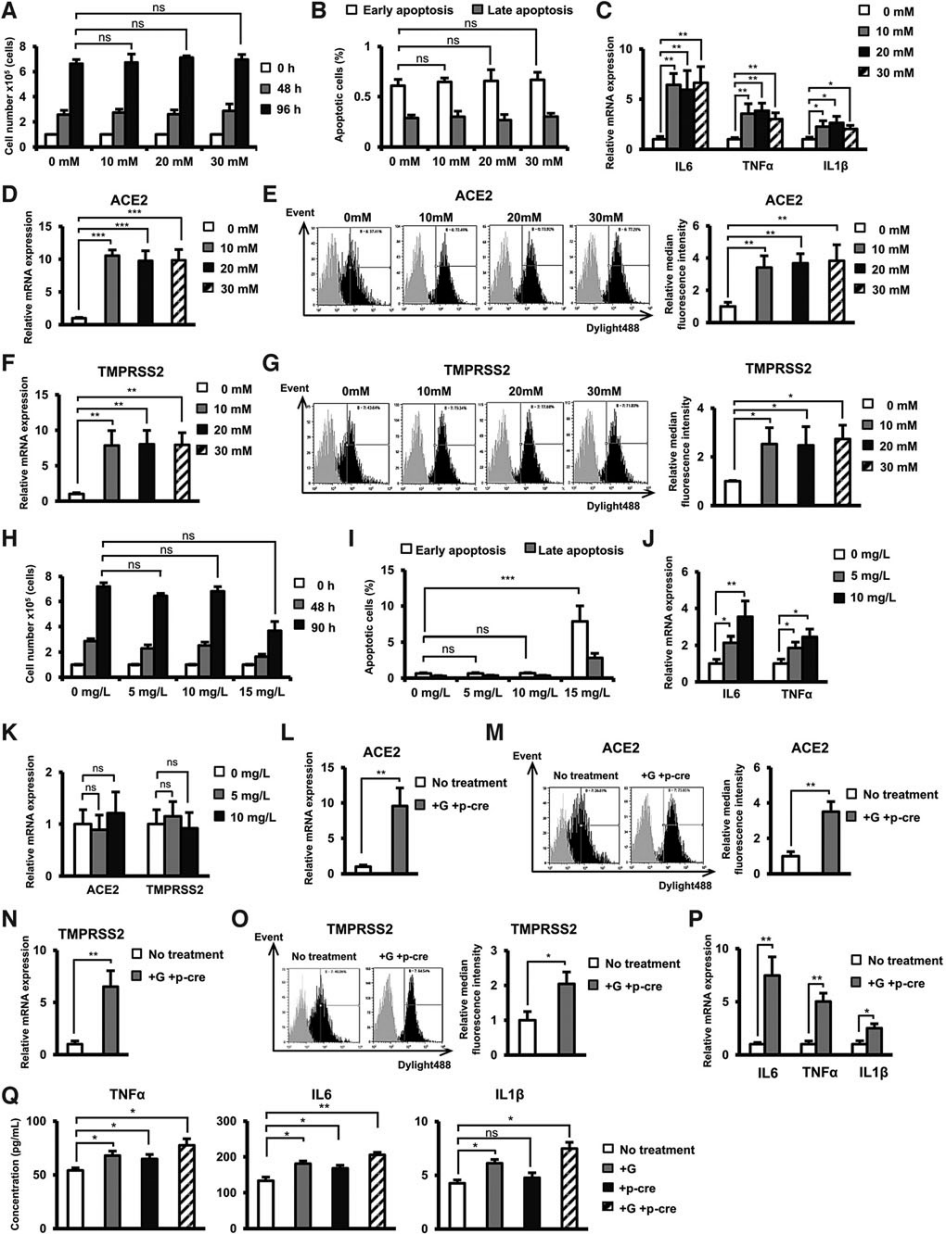
High glucose or/and uremic toxin concentrations induced the expression of inflammatory cytokines in Calu-3 cells. **(A)** The proliferation of Calu-3 cells under high glucose concentrations. **(B)** The number of apoptotic cells under high glucose concentrations. **(C)** The gene expression of inflammatory cytokines in Calu-3 cells under high glucose concentrations. **(D)** The gene expression of ACE2 in Calu-3 cells under high glucose concentrations. Calu-3 was treated with 10, 20, and 30 mM glucose for 24 h. **(E)** The surface expression of ACE2 in Calu-3 cells under high glucose concentrations. **(F)** The gene expression of TMPRSS2 in Calu-3 cells under high glucose concentrations. **(G)** The surface expression of TMPRSS2 in Calu-3 cells under high glucose concentrations. **(H)** The proliferation of Calu-3 cells in the presence of p-cresol as an uremic toxin. **(I)** The number of apoptotic cells in the presence of p-cresol. **(J)** The gene expression of inflammatory cytokines in Calu-3 cells in the presence of p-cresol. **(K)** The gene expression of ACE2 in Calu-3 cells in the presence of p-cresol. Calu-3 cells were cultured under 5 or 10 mg/L p-cresol for 24 h. **(L)** The gene expression of ACE2 in Calu-3 cells in the presence of glucose and p-cresol. **(M)** The surface expression of ACE2 in Calu-3 cells in the presence of glucose and p-cresol. **(N)** The gene expression of TMPRSS2 in Calu-3 cells in the presence of glucose and p-cresol. **(O)** The surface expression of ACE2 in Calu-3 cells in the presence of glucose and p-cresol. **(P)** The gene expression of inflammatory cytokines in Calu-3 cells in the presence of glucose and p-cresol. Three independent experiments were performed for the above experiments (*n* = 3). **(Q)** The secretion of inflammatory cytokines in Calu-3 cells in the presence of glucose and p-cresol. (*n* = 4). Calu-3 cells were cultured under 30 mM Glucose and 10 mg/L p-cresol after 24 h. G: Glucose, p-cre: p-cresol. The data represent the mean ± SD. ***P < 0.001, **P < 0.01, *P < 0.05, ns: no significance. The experiments were performed in triplicate.

The addition of glucose (concentration 10–30 mM) was not associated with the alteration of proliferation or apoptosis of Calu-3 cells (Fig. 4A, B). However, in comparison to cells cultured under normal conditions, a high glucose concentration (10–30 mM) induced the expression of inflammatory cytokines, including TNFα, IL6, and IL1β, (10 mM Glucose: TNFα: 3.5-fold increase, IL6: 6.4-fold increase, and IL1β: 2.2-fold increase; 20 mM Glucose: TNFα: 3.8-fold increase, IL6: 5.9-fold increase, and IL1β: 2.6-fold increase; and 30 mM Glucose: TNFα: 3.2-fold increase, IL6: 6.6-fold increase, and IL1β: 2.1-fold increase, *n* = 3, *P < 0.05, **P < 0.01, Fig. 4C).

Of note, the mRNA expression of ACE2 and TMPRSS2 in Calu-3 cells cultured under high concentrations of glucose was upregulated in comparison to those cultured under normal conditions (ACE2, 10 mM Glucose: 10.2-fold increase, 20 mM Glucose: 9.7-fold increase, 30 mM Glucose: 9.9-fold increase and TMPRSS2, 10 mM Glucose: 7.8-fold increase, 20 mM Glucose: 8.0-fold increase, 30 mM Glucose: 7.9-fold increase *n* = 3, **P < 0.01, ***P < 0.001, Fig. 4D, F). Consistent with the mRNA expression, Calu-3 cells cultured under high concentrations of glucose showed the increased surface expression of ACE2 and TMPRSS2 protein compared to those cultured under normal conditions (ACE2, 10 mM Glucose: 3.4-fold increase, 20 mM Glucose: 3.6-fold increase; and 30 mM Glucose: 3.8-fold increase and TMPRSS2, 10 mM Glucose: 2.5-fold increase, 20 mM Glucose: 2.4-fold increase; and 30 mM Glucose: 2.7-fold increase, *n* = 3, *P < 0.05, **P < 0.01, Fig. 4E, G).

Next, the effects of uremic toxins on Calu-3 cells treated with p-cresol were examined. No significant effects on the proliferation and apoptosis of Calu-3 cells were observed with low concentrations of p-cresol (5 mg/L or 10 mg/L), while an increased concentration of p-cresol (15 mg/L) inhibited the proliferation and induced the apoptosis of the cells (Fig. 4H, I). In addition, treatment with p-cresol at 10 mg/L significantly induced the expression of TNFα and IL6 (TNFα: 2.4-fold increase and IL6: 3.5-fold increase, *n* = 3, *P < 0.05, **P < 0.01, Fig. 4J) in Calu-3 cells. On the other hand, p-cresol showed no significant effect on the expression of ACE2 and TMPRSS2 (Fig. 4K).

Of note, the combination of both glucose and p-cresol treatment resulted in the significant upregulation of ACE2 (9.5-fold increase at mRNA level and 3.5-fold increase at protein level, *n* = 3, *P* < 0.01, Fig. 4L, M) and TMPRSS2 (6.5-fold increase at mRNA level and 2.1-fold increase at protein level, *n* = 3, *P < 0.05, **P < 0.01, Fig. 4N, O), similar to what was observed in Calu-3 cells cultured under a high glucose concentration. In addition, under high glucose and uremic toxin, Calu-3 cells showed the induced expression of inflammatory cytokines, such as TNFα, IL6, and IL1β (TNFα: 5-fold increase, IL6: 7.5-fold increase, and IL1β: 2.5-fold increase, *n* = 3, *P < 0.05, **P < 0.01, Fig. 4P) at the mRNA level and the induced secretion of inflammatory cytokines at the protein level (TNFα: 1.4-fold increase, IL6: 1.5-fold increase, and IL1β: 1.8-fold increase, *n* = 4, *P < 0.05, **P < 0.01, Fig. 4Q).

Taken together, these data indicate that high glucose and p-cresol concentrations affected the upregulation of inflammatory cytokines in Calu-3 cells; meanwhile, only a high glucose concentration induced the expression of ACE2.

### WJ-EVs, but not AT-EVs reduced the SARS-CoV-2 peptide-induced cytokine storm in Calu-3 cells under high glucose and uremic toxin concentrations

**FIG. 5.**
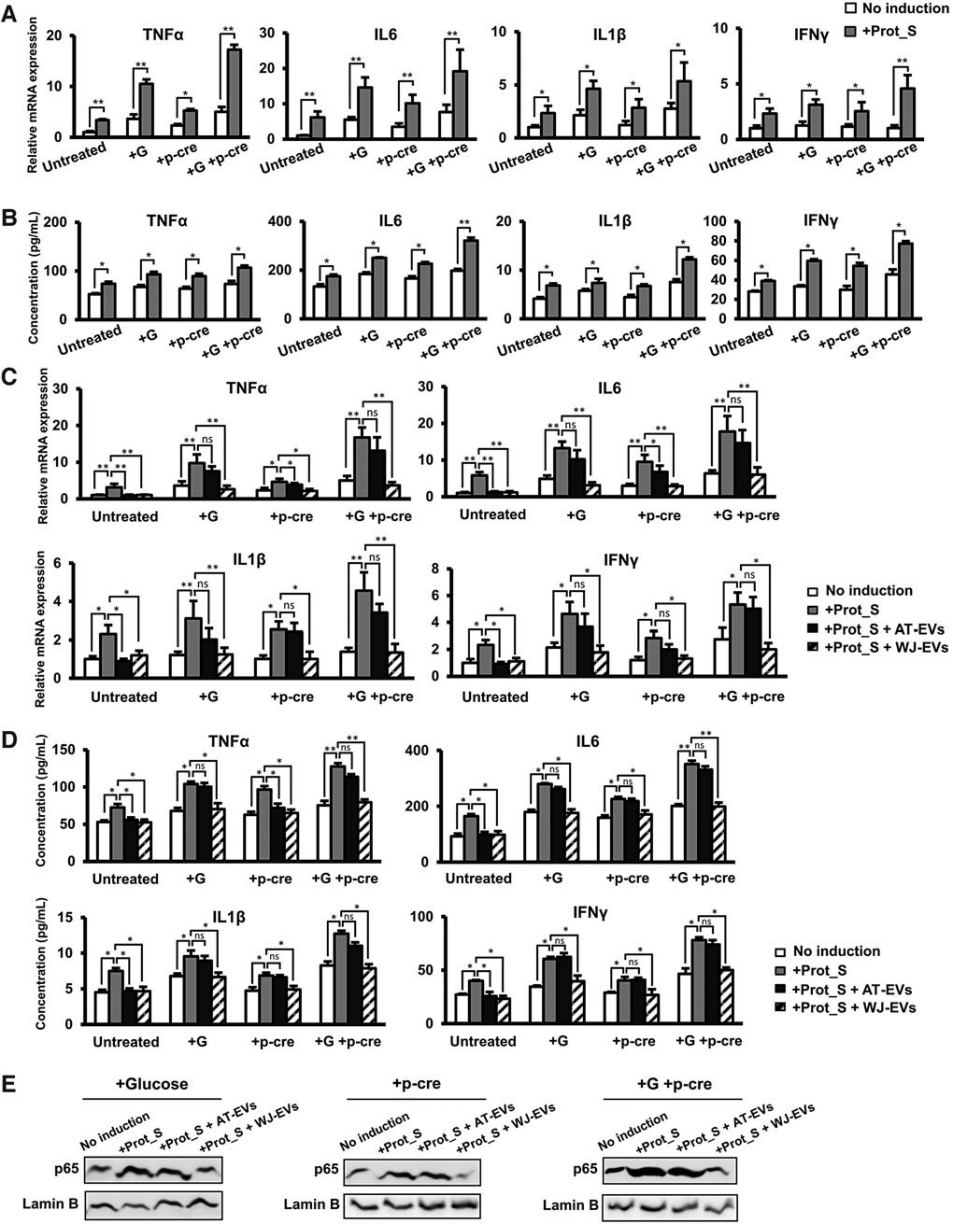
WJ-EVs reduced the cytokine storm of Calu-3 cells induced by SARS-CoV-2 Peptide Pools in high glucose and uremic toxin concentrations. **(A)** The inflammatory cytokine gene expression in Prot_S-induced Calu-3 cells (*n* = 3). **(B)** The inflammatory cytokine secretion in Prot_S-induced Calu-3 cells (*n* = 4). **(C)** The inflammatory cytokine gene expression in Prot_S-induced Calu-3 cells incorporated with EVs. **(D)** The inflammatory cytokine secretion in Prot_S-induced Calu-3 cells incorporated with EVs. **(E)** The nuclear p65 protein expression in Prot_S-induced Calu-3 cells. EVs isolated from four independent cell lines of MSCs were used in the above experiments (*n* = 4). G: Glucose, p-cre: p-cresol. The data represent the mean ± SD. **P < 0.01, *P < 0.05, ns: no significance. The experiments were performed in triplicate. WJ-EV, Wharton's jelly MSC-derived extracellular vesicle.

Next, we examined whether culturing in high glucose and/or uremic toxin concentrations induces inflammatory cytokines of Calu-3 cells with SARS-CoV-2 infection. As expected, culturing in high glucose or uremic toxin concentrations induced the responses of Calu-3 to SARS-CoV-2 Peptide Pools, which exhibited the higher expression and secretion of inflammatory cytokines, including TNFα, IL6, IL1β, and IFNγ (Fig. 5A, B), in comparison to those induced by Prot_S under normal culture conditions. In addition, in comparison to single effects of either glucose or p-cresol, the culture in both high glucose and uremic toxin concentration showed the higher induction of cytokine storm such as TNFα, IL6, IL1β, and IFNγ in Calu-3 cells by Prot_S in both mRNA expression and secreted protein levels (Fig. 5A, B).

We next examined whether AT-EVs and WJ-EVs affect Prot_S-induced inflammatory cytokines in Calu-3 cells cultured under high glucose conditions and/or in the presence of uremic toxin. After induction by Prot_S under high glucose and/or uremic toxin concentrations, the incorporation of either AT-EVs or WJ-EVs into Calu-3 cells was conducted. Notably, the incorporation of AT-EVs into Prot_S-induced Calu-3 cells cultured under high glucose and/or uremic toxin concentrations showed no downregulation of the expression and secretion of inflammatory cytokines (Fig. 5C). In contrast, the incorporation of WJ-EVs significantly reduced the expression of inflammatory cytokines in Calu-3 cells induced by Prot_S, even under high glucose and/or uremic toxin concentrations (high glucose concentration: TNFα: 2.8-fold decrease, IL6: 4.2-fold decrease, IL1β: 2.6-fold decrease, and IFNγ: 2.7-fold decrease; high uremic toxin concentration: TNFα: 2.2-fold decrease, IL6: 3.4-fold decrease, IL1β: 2.5-fold decrease, and IFNγ: 2.2-fold decrease, and high glucose and uremic toxin concentrations: TNFα: 4.6-fold decrease, IL6: 3-fold decrease, IL1β: 3.5-fold decrease, and IFNγ: 2.6-fold decrease, *n* = 4, *P < 0.05, **P < 0.01, Fig. 5C).

Previous studies suggested that the characteristics of cells are affected by the passage number during in vitro culture [36,37]; therefore, we next examined whether EVs from MSCs at a different passage number show similar functions to reduce Prot_S-induced inflammatory cytokines in Calu-3 cells. As shown in Supplementary Fig. S1, EVs isolated from MSCs at passage number 2–8 showed similar effects in which WJ-EVs, but not AT-EVs attenuate the expression of inflammatory cytokines in Prot_S-induced Calu-3 cells under high glucose and uremic toxin conditions.

Next, we measured the concentration of inflammatory cytokines in Prot_S-induced Calu-3 cells. Consistent with the mRNA expression results, the incorporation of WJ-EVs significantly reduced the secretion of inflammatory cytokines in Calu-3 cells induced by Prot_S under high glucose and/or uremic toxin concentrations (high glucose concentration: TNFα: 1.4-fold decrease, IL6: 1.5-fold decrease, IL1β: 1.5-fold decrease, and IFNγ: 1.6-fold decrease; high uremic toxin concentration: TNFα: 1.5-fold decrease, IL6: 1.4-fold decrease, IL1β: 1.4-fold decrease, and IFNγ: 1.5-fold decrease, and high glucose and uremic toxin concentrations: TNFα: 1.6-fold decrease, IL6: 1.8-fold decrease, IL1β: 1.4-fold decrease, and IFNγ: 1.6-fold decrease, *n* = 4, *P < 0.05, **P < 0.01, Fig. 5D).

Moreover, the incorporation of WJ-EVs resulted in the downregulation of nuclear NF-κB p65 in Prot_S-induced Calu-3 cells cultured under high glucose and/or uremic toxin concentrations, while the incorporation of AT-EVs did not result in the downregulation of p65 (high glucose concentration: 2.7-fold decrease, high uremic toxin concentration: 2.5-fold decrease, and high glucose and uremic toxin concentrations, 2.9-fold decrease, *n* = 4, *P* < 0.05, Fig. 5E).

**FIG. 6.**
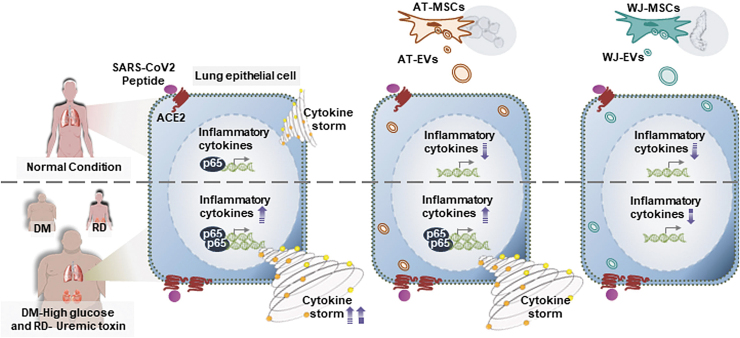
Proposed model: The ability of WJ-EVs in the downregulation of cytokine storm induced by SARS-CoV2 under high concentrations of glucose and uremic toxins. SARS-CoV-2 Peptide induces the cytokine storm, which might be involved in the activation of NF-κB p65 in human lung adenocarcinoma epithelial cells. In patients with DM and RD, the high concentrations of glucose and uremic toxins contribute to the severe cytokine storm induction by SARS-CoV2. WJ-EVs, but not AT-EVs, show the ability to reduce the NF-κB p65 and suppress the cytokine storm induced by SARS-CoV2 in the extraordinary worse situation as high glucose and uremic toxin level.

Taken together, these data suggested that high glucose and/or uremic toxins contributed to the induction of the cytokine storm by Prot_S in Calu-3 cells (Fig. 6). Of note, we found different effects of AT-EVs and WJ-EVs on the altered cytokine expression levels of Prot_S-induced Calu-3 cells cultured under high glucose and/or uremic toxin concentrations. In fact, in comparison to AT-EVs, WJ-EVs showed a significant suppressive effect on cytokine storm, as evidenced by the levels of TNFα, IL6, IL1β, and IFNγ, and the nuclear protein level of NF-κB p65, which were severely altered by Prot_S in Calu-3 cells cultured under high concentrations of glucose and uremic toxins (Fig. 6).

## Discussion

In this study, our data showed that the induction of Prot_S, a spike protein of SARS-CoV-2, altered the cytokine profile of Calu-3 cells, including TNFα, IL6, IL1β, and IFNγ. The upregulation of TNFα, IL6, IL1β, and IFNγ was reported as inflammatory cytokines associated with the postviral infection, which played a role in the recruitment of more immune cells for defense against the virus [38,39]. On the other hand, the induction of these inflammatory cytokines is also recognized as a double-edged sword, in that, the overreaction of inflammatory reaction also contributes to cytokine storm syndrome, leading to organ failure [38]. Our data demonstrated that after being induced by Prot_S, Calu-3 cells showed the upregulation of TNFα, IL6, IL1β, and IFNγ, which is in line with the clinical situation of patients with COVID-19 [38,39].

Moreover, our data also suggested the role of NF-κB p65 in the induction of inflammatory cytokines in Calu-3 cells in response to SARS-CoV2 peptide. Previous studies reported that the binding of SARS-CoV-2 spike protein with ACE2 promotes the hyperinflammatory responses [40]. In this study, we found that induction of Calu-3 cells with SARS-CoV-2 pepTivator Peptide S, which cover the immunodominant sequence domains of spike protein S, resulted in the increased nuclear level of NF-κB p65 protein. In addition, the Prot_S-induced Calu-3 cells after being treated with NF-κB inhibitor showed the reduced nuclear expression of p65, which might be involved in the downregulation of inflammatory cytokines. Our data suggested the responses of Calu-3 cells to SARS-CoV-2 in which the binding of spike glycoprotein S to ACE2 induces the expression of p65, which activated the NF-κB-induced cytokine storm.

Consistent with our finding, another study reported the induction of nuclear translocation of NF-κB p65 in lung carcinoma epithelial cells (A549) infected by SARS-CoV2 [41]. NF-κB has been examined as the mediator that translocates to the nucleus and induces the expression of numerous inflammatory genes, as well as IL6, IL1β, IL8, and IL12 [31]. Therefore, the suppression of NF-κB p65 or the cytokine storm has been considered an appropriate approach for COVID-19 treatment [42,43]. Notably, among the proinflammatory cytokines, IL6 is reported to potentially play an important role in the initiation of the cytokine storm; and the administration of tocilizumab, an anti-IL6 monoclonal antibody, resulted in the improvement of the outcomes of COVID-19 patients, with all patients discharged an average of 15.1 days after the administration of tocilizumab [44].

In the effort to find an effective treatment for COVID-19, several research groups have suggested the application of MSCs due to the immune-modulation ability of these adult stem cells. Several promising outcomes have been reported after the transplantation of ACE2(-) MSCs in patients with COVID-19, including a decreased level of TNFα, an increase in peripheral lymphocytes, the disappearance of overactivated cytokine-secreting immune cells, and the improvement of the pulmonary function [15]. In the light of the immunomodulatory effects of MSCs on SARS-CoV2-infected lung cells, our data demonstrated that both MSCs derived from adipose tissue and those derived from Wharton's jelly clearly showed the ability to downregulate the expression of inflammatory cytokines, such as TNFα, IL6, IL1β, and IFNγ, in Calu-3 cells induced to the normal state by Prot_S (Fig. 3D).

Notably, in addition to MSCs, numerous studies have suggested the application of MSC-EVs as a cell-free therapy for viral infectious diseases [19,21,45]. Actually, a recent study reported the ability of exosomes from bone marrow-derived MSCs to improve the outcomes of patients with COVID-19 based on the ability to induce oxygenation, downregulate the cytokine storm, and induce the recovery of the immune system [21]. Consistently, in our data, the incorporation of either adipose tissue-derived or WJ-derived MSC-EVs into Prot_S-induced Calu-3 cells significantly reduced the altered expression and secretion of inflammatory cytokines, suggesting that MSC-EVs might serve as a promising candidate for the treatment of COVID-19.

It has been reported that some background health conditions, including DM and RD, increase the risk of mortality among COVID-19 patients. Meta-analysis studies have reported the increased risk of mortality in COVID-19 patients with DM [46,47] or RD [47]. Our study showed that COVID-19 patients with both RD and DM had even higher odds of mortality in comparison to those with RD or DM alone. Supporting our results, a study from Mexico reported that the risk of mortality in COVID-19 patients is increased along with an increase in the number of comorbidities [48]. Interestingly, our data showed that under a high glucose concentration in the presence of uremic toxin, Calu-3 cells showed the induction of inflammatory cytokines.

Notably, in response to the induction of SARS-CoV2 Prot_S, the upregulation of inflammatory cytokines was higher in comparison to cells cultured under normal conditions. As a high glucose concentration is associated with DM pathology, while uremic toxin is associated with RD, our data suggested that glucose and uremic toxins contribute to the overreaction of cytokine storm to SARS-CoV2 in patients with chronic inflammatory diseases such as DM, RD, or diabetic nephropathy. Therefore, there is an urgent need to develop an appropriate and effective treatment to reduce the cytokine storm in COVID-19 patients with DM and RD.

Moreover, in view of the severe outcomes of COVID-19 patients with DM, our data also suggested the role of a high glucose concentration in the upregulation of ACE2, an entry receptor of SARS-CoV2. Our data are consistent with previous reports on the induction of ACE2 by high glucose concentrations in several cell types, including vascular smooth muscle cells and pancreatic β-cells [49–51]. In addition, another study also showed the higher protein expression of ACE2 in the lungs of DM patients in comparison to those without DM, suggesting that DM patients might have a higher risk of SARS-CoV-2 infection [52]. Therefore, it is necessary to develop specific treatments to prevent the risk of COVID-19 infection in DM patients.

## Conclusions

Taken together, this study suggested that high concentrations of glucose and uremic toxins might contribute to the overreaction of the cytokine storm induced by SARS-CoV2 infection, thus resulting in the more severe outcomes and higher mortality in patients with diseases associated with chronic inflammation, such as DM and RD. Of note, our data also clearly showed that using WJ-EVs would be one of the best immunomodulation therapies for treating the SARS-CoV2-induced cytokine storm in patients with DM and RD. In addition, it is worth clarifying the efficiency of using WJ-EVs in the treatment of other infectious diseases in the near future.

## Supplementary Material

Supplemental data

## References

[1] Shang J, Y Wan, C Luo, G Ye, Q Geng, A Auerbach and F Li. (2020). Cell entry mechanisms of SARS-CoV-2. Proc Natl Acad Sci USA 117:11727– 11734 32376634 10.1073/pnas.2003138117 PMC7260975

[2] Merad M and JC Martin. (2020). Pathological inflammation in patients with COVID-19: a key role for monocytes and macrophages. Nat Rev Immunol 20:355–362 32376901 10.1038/s41577-020-0331-4 PMC7201395

[3] Acharya D, G Liu and MU Gack. (2020). Dysregulation of type I interferon responses in COVID-19. Nat Rev Immunol 20:397–398 32457522 10.1038/s41577-020-0346-x PMC7249038

[4] Liu Q, Y Zhou and Z Yang. (2016). The cytokine storm of severe influenza and development of immunomodulatory therapy. Cell Mol Immunol 13:3–10 26189369 10.1038/cmi.2015.74 PMC4711683

[5] Bhaskar S, A Sinha, M Banach, S Mittoo, R Weissert, JS Kass, S Rajagopal, AR Pai and S Kutty. (2020). Cytokine Storm in COVID-19-Immunopathological Mechanisms, Clinical Considerations, and Therapeutic Approaches: the REPROGRAM Consortium Position Paper. Front Immunol 11:1648 32754159 10.3389/fimmu.2020.01648 PMC7365905

[6] Barron E, C Bakhai, P Kar, A Weaver, D Bradley, H Ismail, P Knighton, N Holman, K Khunti, et al. (2020). Associations of type 1 and type 2 diabetes with COVID-19-related mortality in England: a whole-population study. Lancet Diabetes Endocrinol 8:813–822 32798472 10.1016/S2213-8587(20)30272-2 PMC7426088

[7] Bode B, V Garrett, J Messler, R McFarland, J Crowe, R Booth and DC Klonoff. (2020). Glycemic characteristics and clinical outcomes of COVID-19 patients hospitalized in the United States. J Diabetes Sci Technol 14:813–821 32389027 10.1177/1932296820924469 PMC7673150

[8] Huang I, MA Lim and R Pranata. (2020). Diabetes mellitus is associated with increased mortality and severity of disease in COVID-19 pneumonia - A systematic review, meta-analysis, and meta-regression. Diabetes Metab Syndr 14:395–403 32334395 10.1016/j.dsx.2020.04.018 PMC7162793

[9] Apicella M, MC Campopiano, M Mantuano, L Mazoni, A Coppelli and S Del Prato. (2020). COVID-19 in people with diabetes: understanding the reasons for worse outcomes. Lancet Diabetes Endocrinol 8:782–792 32687793 10.1016/S2213-8587(20)30238-2 PMC7367664

[10] Gansevoort RT and LB Hilbrands. (2020). CKD is a key risk factor for COVID-19 mortality. Nat Rev Nephrol 16:705–706 32848205 10.1038/s41581-020-00349-4 PMC7447963

[11] Husain SA, G Dube, H Morris, H Fernandez, J-H Chang, K Paget, S Sritharan, S Patel, O Pawliczak, et al. (2020). Early outcomes of outpatient management of kidney transplant recipients with coronavirus disease 2019. Clin J Am Soc Nephrol 15:1174–1178 32423908 10.2215/CJN.05170420 PMC7409755

[12] Wu J, J Li, G Zhu, Y Zhang, Z Bi, Y Yu, B Huang, S Fu, Y Tan, J Sun and X Li. (2020). Clinical Features of Maintenance Hemodialysis Patients with 2019 Novel Coronavirus-Infected Pneumonia in Wuhan, China. Clin J Am Soc Nephrol 15:1139–1145 32444393 10.2215/CJN.04160320 PMC7409740

[13] Ajaimy M and ML Melamed. (2020). COVID-19 in Patients with Kidney Disease. Clin J Am Soc Nephrol 15:1087–1089 32636199 10.2215/CJN.09730620 PMC7409763

[14] Song N, M Scholtemeijer and K Shah. (2020). Mesenchymal stem cell immunomodulation: mechanisms and therapeutic potential. Trends Pharmacol Sci 41:653–664 32709406 10.1016/j.tips.2020.06.009 PMC7751844

[15] Leng Z, R Zhu, W Hou, Y Feng, Y Yang, Q Han, G Shan, F Meng, D Du, et al. (2020). Transplantation of ACE2(-) mesenchymal stem cells improves the outcome of patients with COVID-19 Pneumonia. Aging Dis 11:216–228 32257537 10.14336/AD.2020.0228 PMC7069465

[16] Schäfer R, G Spohn, M Bechtel, D Bojkova, PC Baer, S Kuçi, E Seifried, S Ciesek and J Cinatl. (2020). Human mesenchymal stromal cells are resistant to SARS-CoV-2 Infection under Steady-State, Inflammatory Conditions and in the Presence of SARS-CoV-2-Infected Cells. Stem Cell Reports 16:419–427 32950067 10.1016/j.stemcr.2020.09.003 PMC7486048

[17] Baek G, H Choi, Y Kim, H-C Lee and C Choi. (2019). Mesenchymal stem cell-derived extracellular vesicles as therapeutics and as a drug delivery platform. Stem Cells Transl Med 8:880–886 31045328 10.1002/sctm.18-0226 PMC6708072

[18] Keshtkar S, N Azarpira and MH Ghahremani. (2018). Mesenchymal stem cell-derived extracellular vesicles: novel frontiers in regenerative medicine. Stem Cell Res Ther 9:63 29523213 10.1186/s13287-018-0791-7 PMC5845209

[19] Seo Y, H-S Kim and I-S Hong. (2019). Stem cell-derived extracellular vesicles as immunomodulatory therapeutics. Stem Cells Int 2019:5126156 30936922 10.1155/2019/5126156 PMC6413386

[20] Qian X, C Xu, S Fang, P Zhao, Y Wang, H Liu, W Yuan and Z Qi. (2016). Exosomal MicroRNAs derived from umbilical mesenchymal stem cells inhibit hepatitis C virus infection. Stem Cells Transl Med 5:1190–1203 27496568 10.5966/sctm.2015-0348 PMC4996444

[21] Sengupta V, S Sengupta, A Lazo, P Woods, A Nolan and N Bremer. (2020). Exosomes derived from bone marrow mesenchymal stem cells as treatment for severe COVID-19. Stem Cells Dev 29:747–754 32380908 10.1089/scd.2020.0080 PMC7310206

[22] Kimura K, M Nagano, G Salazar, T Yamashita, I Tsuboi, H Mishima, S Matsushita, F Sato, K Yamagata and O Ohneda. (2013). The Role of CCL5 in the ability of adipose tissue-derived mesenchymal stem cells to support repair of ischemic regions. Stem Cells Dev 23:488–501 24171667 10.1089/scd.2013.0307 PMC3928761

[23] Schneider CA, WS Rasband and KW Eliceiri. (2012). NIH Image to ImageJ: 25years of image analysis. Nat Methods 9:671–675 22930834 10.1038/nmeth.2089 PMC5554542

[24] Mihai S, E Codrici, ID Popescu, A-M Enciu, L Albulescu, LG Necula, C Mambet, G Anton and C Tanase. (2018). Inflammation-related mechanisms in chronic kidney disease prediction, progression, and outcome. J Immunol Res 2018:2180373 30271792 10.1155/2018/2180373 PMC6146775

[25] Harcourt JL, H Caidi, LJ Anderson and LM Haynes. (2011). Evaluation of the Calu-3cell line as a model of in vitro respiratory syncytial virus infection. J Virol Methods 174:144–149 21458491 10.1016/j.jviromet.2011.03.027 PMC7112923

[26] Hoffmann M, H Kleine-Weber, S Schroeder, N Krüger, T Herrler, S Erichsen, TS Schiergens, G Herrler, N-H Wu, et al. (2020). SARS-CoV-2 Cell entry depends on ACE2 and TMPRSS2 and is blocked by a clinically proven protease inhibitor. Cell 181:271–280.e8. 32142651 10.1016/j.cell.2020.02.052 PMC7102627

[27] Sheahan TP, AC Sims, S Zhou, RL Graham, AJ Pruijssers, ML Agostini, SR Leist, A Schäfer, KH 3rd Dinnon, et al. (2020). An orally bioavailable broad-spectrum antiviral inhibits SARS-CoV-2 in human airway epithelial cell cultures and multiple coronaviruses in mice. Sci Transl Med 12:eabb5883 32253226 10.1126/scitranslmed.abb5883 PMC7164393

[28] Dittmar M, JS Lee, K Whig, E Segrist, M Li, B Kamalia, L Castellana, K Ayyanathan, FL Cardenas-Diaz, et al. (2021). Drug repurposing screens reveal cell-type-specific entry pathways and FDA-approved drugs active against SARS-Cov-2. Cell Rep 35:108959 33811811 10.1016/j.celrep.2021.108959 PMC7985926

[29] Brownlee M. (2005). The pathobiology of diabetic complications. Diabetes 54:1615–1625 15919781 10.2337/diabetes.54.6.1615

[30] Yamamoto S. (2019). Molecular mechanisms underlying uremic toxin-related systemic disorders in chronic kidney disease: focused on β(2)-microglobulin-related amyloidosis and indoxyl sulfate-induced atherosclerosis-Oshima Award Address 2016. Clin Exp Nephrol 23:151–157 29869756 10.1007/s10157-018-1588-9 PMC6510801

[31] Liu T, L Zhang, D Joo and S-C Sun. (2017). NF-kappaB signaling in inflammation. Signal Transduct Target Ther 2:17023 29158945 10.1038/sigtrans.2017.23 PMC5661633

[32] Gowen A, F Shahjin, S Chand, KE Odegaard and S V Yelamanchili. (2020). Mesenchymal stem cell-derived extracellular vesicles: challenges in clinical applications. Front Cell Dev Biol 8:149 32226787 10.3389/fcell.2020.00149 PMC7080981

[33] Abdul-Ghani MA and RA DeFronzo. (2009). Plasma glucose concentration and prediction of future risk of type 2 diabetes. Diabetes Care 32 Suppl 2:S194–S198 10.2337/dc09-S309 PMC2811468 19875551

[34] Katherine E, N Francesco, M Raffaele, G Giovanni, G Francesco, C Myriam, Q Lisa, C Antonio and G Dario. (2002). Inflammatory cytokine concentrations are acutely increased by hyperglycemia in humans. Circulation 106:2067–2072 12379575 10.1161/01.cir.0000034509.14906.ae

[35] Castillo-Rodríguez E, S Pizarro-Sánchez, AB Sanz, AM Ramos, MD Sanchez-Niño, C Martin-Cleary, B Fernandez-Fernandez and A Ortiz. (2017). Inflammatory Cytokines as Uremic Toxins: “Ni Son Todos Los Que Estan, Ni Estan Todos Los Que Son.” Toxins (Basel) 9:114 10.3390/toxins9040114 PMC5408188 28333114

[36] Legzdina D, A Romanauska, S Nikulshin, T Kozlovska and U Berzins. (2016). Characterization of senescence of culture-expanded human adipose-derived mesenchymal stem cells. Int J Stem Cells 9:124–136 27426094 10.15283/ijsc.2016.9.1.124 PMC4961112

[37] Drela K, L Stanaszek, A Nowakowski, Z Kuczynska and B Lukomska. (2019). Experimental strategies of mesenchymal stem cell propagation: adverse events and potential risk of functional changes. Stem Cells Int 2019:7012692 30956673 10.1155/2019/7012692 PMC6431404

[38] Del Valle DM, S Kim-Schulze, H-H Huang, ND Beckmann, S Nirenberg, B Wang, Y Lavin, TH Swartz, D Madduri, et al. (2020). An inflammatory cytokine signature predicts COVID-19 severity and survival. Nat Med 26:1636–1643 32839624 10.1038/s41591-020-1051-9 PMC7869028

[39] de la Rica R, M Borges and M Gonzalez-Freire. (2020). COVID-19: in the eye of the cytokine storm. Front Immunol 11:558898 33072097 10.3389/fimmu.2020.558898 PMC7541915

[40] Hsu AC-Y, G Wang, AT Reid, PC Veerati, PS Pathinayake, K Daly, JR Mayall, PM Hansbro, JC Horvat, F Wang and PA Wark. (2020). SARS-CoV-2 Spike protein promotes hyper-inflammatory response that can be ameliorated by Spike-antagonistic peptide and FDA-approved ER stress and MAP kinase inhibitors in vitro. BioRxiv [Epub ahead of print]; DOI: 10.1101/2020.09.30.317818

[41] Neufeldt CJ, B Cerikan, M Cortese, J Frankish, J-Y Lee, A Plociennikowska, F Heigwer, S Joecks, SS Burkartet al. (2020). SARS-CoV-2 infection induces a pro-inflammatory cytokine response through cGAS-STING and NF-κB. BioRxiv [Epub ahead of print]; DOI: 10.1101/2020.07.21.212639 PMC8755718 35022513

[42] Hariharan A, AR Hakeem, S Radhakrishnan, MS Reddy and M Rela. (2020). The role and therapeutic potential of NF-kappa-B pathway in severe COVID-19 patients. Inflammopharmacology 29:91–100 33159646 10.1007/s10787-020-00773-9 PMC7648206

[43] Ye Q, B Wang and J Mao. (2020). The pathogenesis and treatment of the `Cytokine Storm’ in COVID-19. J Infect 80:607–613 32283152 10.1016/j.jinf.2020.03.037 PMC7194613

[44] Xu X, M Han, T Li, W Sun, D Wang, B Fu, Y Zhou, X Zheng, Y Yang, et al. (2020). Effective treatment of severe COVID-19 patients with tocilizumab. Proc Natl Acad Sci USA 117:10970–10975 32350134 10.1073/pnas.2005615117 PMC7245089

[45] Khatri M, LA Richardson and T Meulia. (2018). Mesenchymal stem cell-derived extracellular vesicles attenuate influenza virus-induced acute lung injury in a pig model. Stem Cell Res Ther 9:17 29378639 10.1186/s13287-018-0774-8 PMC5789598

[46] Chidambaram V, NL Tun, WZ Haque, MG Majella, RK Sivakumar, A Kumar, AT-W Hsu, IA Ishak, AA Nur, et al. (2020). Factors associated with disease severity and mortality among patients with COVID-19: a systematic review and meta-analysis. PLoS One 15:e0241541 33206661 10.1371/journal.pone.0241541 PMC7673562

[47] Dorjee K, H Kim, E Bonomo and R Dolma. (2020). Prevalence and predictors of death and severe disease in patients hospitalized due to COVID-19: a comprehensive systematic review and meta-analysis of 77 studies and 38,000 patients. PLoS One 15:e0243191 33284825 10.1371/journal.pone.0243191 PMC7721151

[48] Kammar-García A, J de J Vidal-Mayo, JM Vera-Zertuche, M Lazcano-Hernández, O Vera-López, O Segura-Badilla, P Aguilar-Alonso and AR Navarro-Cruz. (2020). IMPACT OF COMORBIDITIES IN MEXICAN SARS-COV-2-POSITIVE PATIENTS: a RETROSPECTIVE ANALYSIS IN A NATIONAL COHORT. Rev Invest Clin 72:151–158 32584330 10.24875/RIC.20000207

[49] Lavrentyev EN and KU Malik. (2008). High glucose (HG)-induced angiotensin-converting enzyme 2 (ACE2) and angiotensin (1–7) [Ang (1–7)] decrease is prevented by captopril, losartan and insulin in rat vascular smooth muscle cells (VSMCs). FASEB J 22:912.18–912.18.

[50] Härdtner C, C Mörke, R Walther, C Wolke and U Lendeckel. (2013). High glucose activates the alternative ACE2/Ang-(1-7)/Mas and APN/Ang IV/IRAP RAS axes in pancreatic β-cells. Int J Mol Med 32:795–804 23942780 10.3892/ijmm.2013.1469 PMC3812297

[51] N. LE, EA M. and MK U. (2007). Mechanism of high glucose–induced Angiotensin II production in rat vascular smooth muscle cells. Circ Res 101:455–464 17626897 10.1161/CIRCRESAHA.107.151852

[52] Wijnant SRA, M Jacobs, HP Van Eeckhoutte, B Lapauw, GF Joos, KR Bracke and GG Brusselle (2020). Expression of ACE2, the SARS-CoV-2 receptor, in lung tissue of patients with type 2 diabetes. Diabetes 69:2691–2699 33024003 10.2337/db20-0669

