# Supplementary material for: Wharton's Jelly Mesenchymal Stem Cell-Derived Extracellular Vesicles Reduce SARS-CoV2-Induced Inflammatory Cytokines Under High Glucose and Uremic Toxin Conditions
Source: Stem Cells Dev. 2021 Aug 2;30(15):758–72. doi: 10.1089/scd.2021.0065 (PMC8356045; doi:10.1089/scd.2021.0065)
Supplement: Supplemental data [file Supp_Fig1.docx]

**Figure Legend**

**
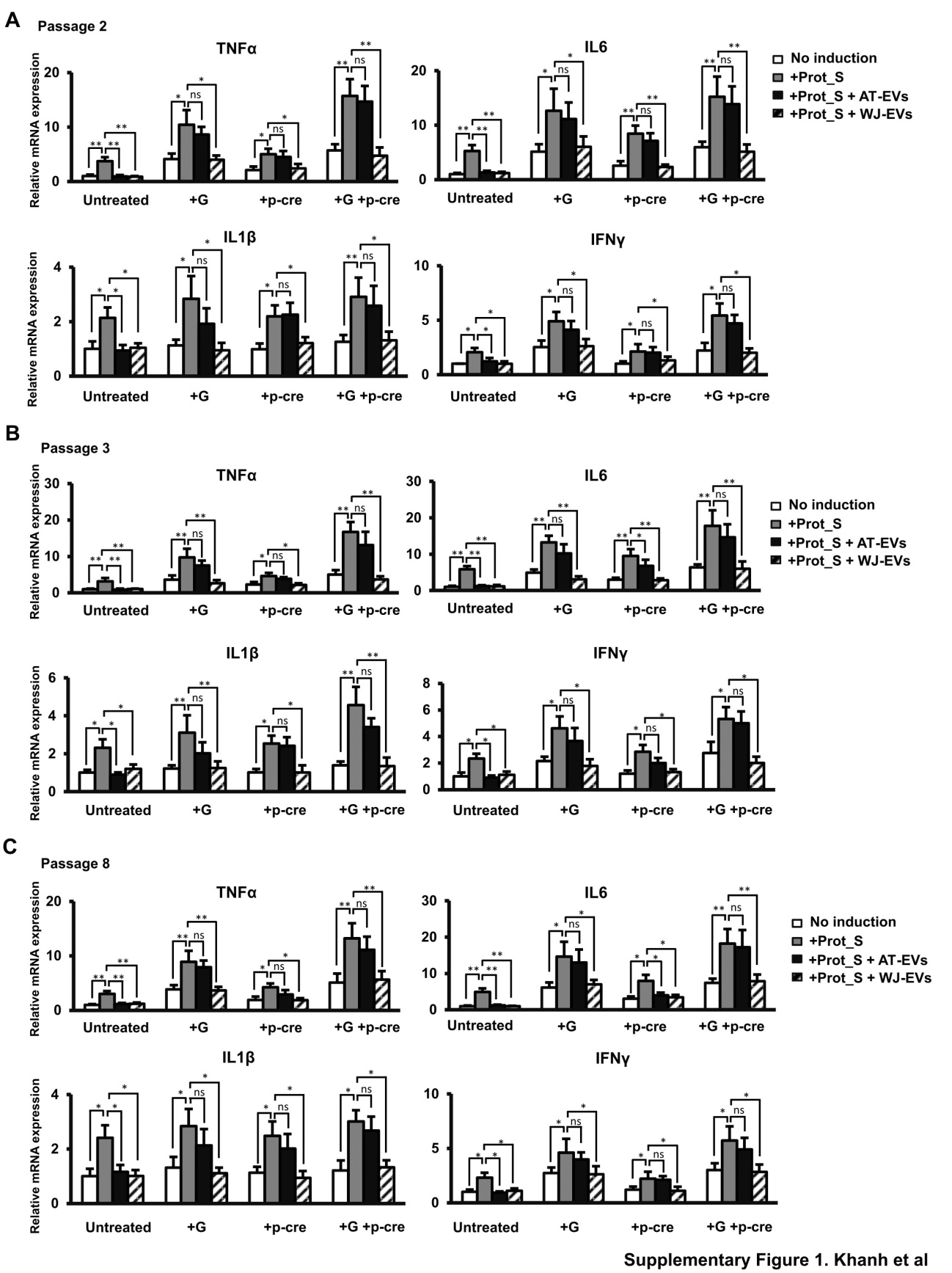
**

**Supplementary Figure 1. The expression of proinflammatory cytokines in Calu-3 cells. A.** The inflammatory cytokine gene expression in Prot_S-induced Calu-3 cells treated with EVs derived from AT-MSCs or WJ-MSCs at passage 2. **B.** The inflammatory cytokine gene expression in Prot_S-induced Calu-3 cells treated with EVs derived from AT-MSCs or WJ-MSCs at passage 3. **C.** The inflammatory cytokine gene expression in Prot_S-induced Calu-3 cells treated with EVs derived from AT-MSCs or WJ-MSCs at passage 8. EVs isolated from four independent cell lines of MSCs were used in the above experiments (n=4). The data represent the mean ± SD. **P<0.01, *P<0.05, ns: no significance. The experiments were performed in triplicate.
